# Adenosine A2a Receptor Stimulation Attenuates Ischemia-Reperfusion Injury and Improves Survival in A Porcine Model of DCD Liver Transplantation

**DOI:** 10.3390/ijms21186747

**Published:** 2020-09-14

**Authors:** Zoltan Czigany, Eve Christiana Craigie, Georg Lurje, Shaowei Song, Kei Yonezawa, Yuzo Yamamoto, Thomas Minor, René Hany Tolba

**Affiliations:** 1 Department of Surgery and Transplantation, Faculty of Medicine, University Hospital RWTH Aachen, 52074 Aachen, Germany; zczigany@ukaachen.de; 2 Institute for Laboratory Animal Science and Experimental Surgery, Faculty of Medicine, RWTH-Aachen University, 52074 Aachen, Germany; eve.craigie@gmail.com; 3 Department of Surgery, Campus Charité Mitte | Campus Virchow-Klinikum–Charité-Universitätsmedizin, 13353 Berlin, Germany; georg.lurje@charite.de; 4 Department of Surgery, The First Affiliated Hospital of China Medical University, Shenyang 110122, China; songshaoweicmu@126.com; 5 Department of Surgery, Shizuoka City Hospital, Shizuoka 420-8527, Japan; vyh06211@nifty.com; 6 Department of Gastroenterological Surgery, Akita University Graduate School of Medicine, Akita 010-0825, Japan; yy@med.akita-u.ac.jp; 7 Department of General, Visceral, and Transplantation Surgery, University Hospital Essen, 45147 Essen, Germany; thomas.minor@uk-essen.de

**Keywords:** adenosine A2a agonist, liver transplantation, microcirculation, porcine model, donation after circulatory death, survival, reperfusion injury, hepatoprotection

## Abstract

Orthotopic liver transplantation (OLT) using allografts from donation after circulatory death (DCD) is potentially associated with compromised clinical outcomes due to ischemia-reperfusion injury (IRI)-induced organ damage and graft-related complications. The aim of this study was to provide in vivo data on the effects of adenosine A_2a_ receptor stimulation in a clinically relevant large animal model of DCD liver transplantation. Cardiac arrest was induced in German Landrace pigs (*n* = 10; 20–25 kg). After 30 min of warm ischemia, the donor liver was retrieved following a cold flush with 3 L of histidine-tryptophan-ketoglutarate-HTK solution. Animals of the treatment group (*n* = 5/group) received a standard dose of the selective adenosine receptor agonist CGS 21680 added to the cold flush. All grafts were stored for 4.5 h at 4 °C in HTK-solution before OLT. Hepatocellular injury, apoptosis, protein kinase A-PKA activity, graft microcirculation, liver function, and animal survival were assessed. Compared to untreated livers, adenosine A_2a_ receptor stimulation resulted in improved tissue microcirculation (103% ± 5% vs. 38% ± 4% compared to baseline; *p* < 0.05), accelerated functional recovery of the graft (indocyanine green-plasma disappearance rate (ICG-PDR) of 75% ± 18% vs. 40% ± 30% after 3 h), increased PKA activity ratio (56% ± 3% vs. 32% ± 3%; *p* < 0.001 after 1 h), and consequently reduced tissue necrosis and apoptosis. The potent protective effects were clinically manifested in significantly improved survival in the treatment group after 72 h (100% vs. 40%; *p =* 0.04). The ex vivo administration of adenosine A_2a_ receptor agonist during the back-table flush mitigates IRI-mediated tissue damage and improves functional graft recovery and survival in a large animal model of DCD liver transplantation.

## 1. Introduction

Since the ground-breaking efforts of the pioneering group of Thomas Starzl in 1963, orthotopic liver transplantation (OLT) has evolved as the treatment of choice for patients with chronic liver disease and acute liver failure [1,2]. Despite many improvements in terms of surgical techniques, peri-operative intensive therapy, and organ preservation, ischemic-reperfusion injury (IRI) still represents a major source of allograft dysfunction and inferior outcomes following OLT [1,2,3,4]. The significance of IRI is further underlined by some fundamental changes in the clinical practice of recent years, due to a critical organ shortage and the consequential need for an expansion of the available donor pool [1,3].

Allografts that would have previously been rejected for organ transplantation due to their inferior quality have nowadays become an important part of the donor pool as marginal or extended criteria donor (ECD) allografts [1,5,6]. Aside from advanced donor age, long cold preservation, or the presence of a significant macrosteatosis, donation after circulatory death (DCD) is one of the most frequently employed features to define marginal ECD allografts [1,6,7]. Although many centers successfully transplant a high number of liver allografts from DCD donors, in the case of these particular livers, the additional period of in situ warm ischemia time during retrieval exposes the recipient to an increased risk of developing graft-related complications, such as delayed graft function-DGF, primary non-function-PNF, or biliary complications, leading to inferior long-term outcomes [8,9,10].

Various innovative methods have been introduced and investigated over the years by our group and by others with the aim of increasing the tolerance of ECD allografts to prolonged periods of warm and cold ischemia and reducing hepatocellular damage following reperfusion. These include the use of (remote) ischemic conditioning [3,11,12,13], machine perfusion and dynamic organ preservation [1,5,14,15,16], modification and enhancement of preservation solutions by cytoprotective agents [17,18], and even “supercooling” [19].

Adenosine receptor activation has been demonstrated to decrease pro-inflammatory cytokine secretion, neutrophil activation, platelet aggregation, and the proliferation of lymphocytes [20,21,22]. The selective stimulation of the adenosine A_2a_ receptor has been shown to be effective in reducing IRI-related damage in different tissues and organs including the liver [22]. The cellular responses of A_2a_ receptor activation seem to be attributed to cyclic adenosine monophosphate (cAMP)- and PKA-dependent pathways [22], resulting in reduced inflammation and reactive oxygen species (ROS) production, as well as mitigated microvascular/endothelial injury and cell death [22,23,24]. Despite its potentially beneficial role in IRI-related graft damage, no in vivo results are available on the effects of selective A_2a_ receptor activation in a large animal model of DCD liver transplantation [25,26,27].

In this study, we aimed to evaluate the specific in vivo effects of the adenosine A_2a_ receptor agonist CGS 21680 on IRI, graft viability, and survival in a clinically relevant porcine model of DCD liver transplantation.

## 2. Results

### 2.1. Microcirculation

**Figure 1:**
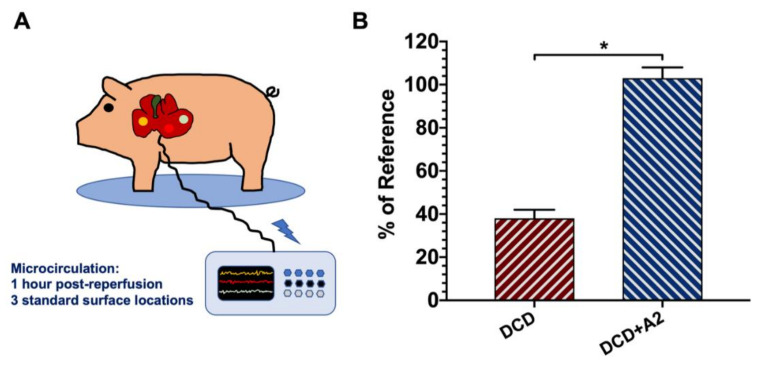
Hepatic microcirculation. (**A**) Graft microcirculation was evaluated one hour after reperfusion at three standard locations on the surface of the liver using a laser Doppler monitor and corresponding surface probe. The mean value of the measurement points was used to characterize graft microcirculation. (**B**) Due to the large individual variations between animals, the data were expressed as a percentage of the baseline value (mean ± standard error of the mean (s.e.m.), * *p* < 0.05 donation after circulatory death (DCD) + A2 vs. DCD, Student’s t-test, *n* = 5/group).

Although the microcirculation data of the DCD + A2 group stayed around the preischemic baseline values, there was a prominent decrease in microcirculation and tissue perfusion in the DCD group, with significant between-group differences (103% ± 5% vs. 38% ± 4%, DCD + A2 vs. DCD; *p* < 0.05; see Figure 1).

### 2.2. Allograft Functional Recovery

**Figure 2:**
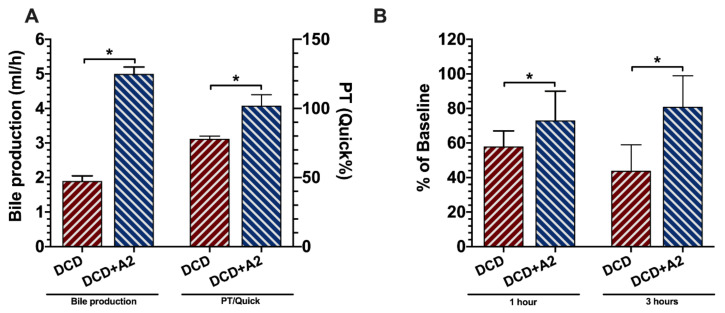
Graft functional recovery. (**A**) Bile production is presented as total amount of bile collected over the 1st hour of reperfusion in the DCD and DCD + A2 groups. Prothrombin time (PT)/Quick values at post-operative day 3 (POD3) following orthotopic liver transplantation (OLT) in the DCD and DCD + A2 groups. (**B**) Indocyanine green (ICG) plasma disappearance rate was measured in the DCD and DCD + A2 groups at 1 h and 3 h after reperfusion and presented as a percentage of ICG eliminated compared to baseline. (mean ± s.e.m., * *p* < 0.05 DCD + A2 vs. DCD, Student’s *t*-test, *n* = 5/group).

Bile production, as an important parameter of early graft function and functional recovery, was measured over the first hour of reperfusion. Bile production in the DCD control liver was 1.90 ± 0.15 mL/h. The treatment with A_2a_ receptor agonist resulted in an improved bile production with 5.00 ± 0.20 mL in the DCD + A2 group (*p* < 0.05, Figure 2).

To further assess the various aspects of graft function, prothrombin time (PT) was measured on post-operative day 3 (POD3) following OLT. After three days, normal coagulation was observed in the DCD + A2 group with a PT of 102% ± 8% in Quick’s method in comparison to the significantly reduced levels in the untreated surviving animals (vs. 78% ± 2%; *p* < 0.05, see Figure 2).

**Figure 3:**
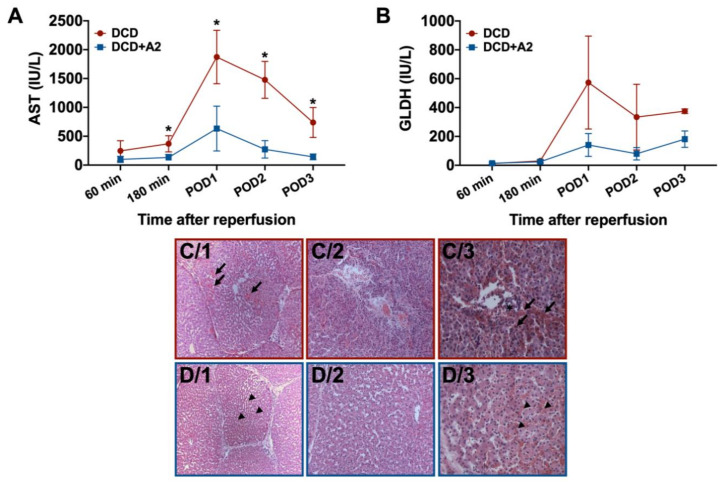
Hepatocellular damage and histopathology. (**A**) Time course of serum aspartate-aminotransferase (AST) levels expressed in IU/L in the DCD and DCD + A2 groups (mean ± s.e.m., * *p* < 0.05 DCD + A2 vs. DCD, Student’s *t*-test, *n* = 5/group) (**B**) Time course of serum glutamate dehydrogenase (GLDH) levels expressed in IU/L in the DCD and DCD + A2 groups (mean ± s.e.m., *n* = 5/group (**C1**–**3**) Representative microscopic images of the histological specimens at sacrifice in the DCD group (stained with hematoxylin-eosin; original magnification 100×, 200×, 400×, respectively). (**D1**–**3**) Representative microscopic images of the histological specimens at sacrifice in the DCD + A2 group (stained with hematoxylin-eosin; original magnification 100×, 200×, 400×, respectively).

The 30 min of circulatory arrest and 4.5 h of cold storage resulted in a prominent reduction of graft function in both groups, as characterized by the ICG-PDR at 1 and 3 h following reperfusion. After 1 hour, the liver’s clearance function was 58% ± 9% in the DCD group, whereas significantly more ICG was cleared by the liver in the DCD + A2 group with 73% ± 17% (*p* < 0.05, see Figure 3). Even more pronounced functional deterioration was observed in the DCD control animals following 3 h of reperfusion, whereas an improvement of allograft clearance capacity was documented in the treated DCD + A2 group (44% ± 15% vs. 81% ± 18%; *p* < 0.05, see Figure 2).

### 2.3. Hepatocellular Injury and Apoptosis

In the untreated DCD group, 60% to 80% of the examined visual fields showed various degrees of injury after three days, including moderate to severe hepatocyte degeneration, necrosis, sinusoidal congestion, and hemorrhage. In the portal areas, bile ducts also showed damage of the biliary epithelial lining, including disintegration of the surrounding connective tissue (Figure 3). In addition, a mild to moderate increase of neutrophil presence was observed in the sinusoids.

Similar lesions, although a milder injury in general, were observed on the slides of the DCD + A2 groups, with approximately 25–30% of the visual fields and lobuli showing a relevant level of damage (Figure 3). In line with the histopathological findings, hepatocellular damage, monitored by the measurement of serum aspartate aminotransferase (AST), increased prominently, reaching its peak on POD1 in both experimental groups. However, an almost 3-fold higher peak was observed in the untreated DCD group compared to the DCD + A2 animals (1873 ± 463 IU/L vs. 633 ± 388 IU/L; *p* < 0.05, see Figure 3). Subsequently, AST levels decreased gradually until sacrifice. On POD3, the DCD group still presented markedly increased AST levels compared to the significantly lowered AST values measured in the DCD + A2 group (738 ± 259 IU/L vs. 143 ± 50 IU/L; *p <* 0.05, see Figure 3).

**Figure 4:**
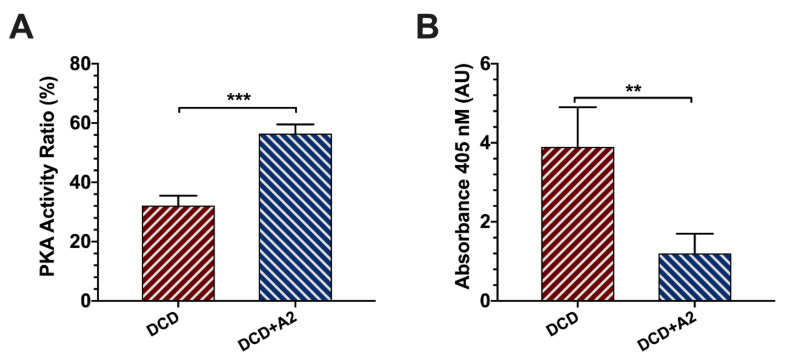
PKA activation and corresponding DNA fragmentation and apoptosis. (**A**) Serum activity of protein kinase A (PKA) after 1 hour of reperfusion shown in activity ratio in the DCD and DCD + A2 groups. (mean ± s.e.m., *** *p* < 0.001 DCD + A2 vs. DCD, Student’s t-test, *n* = 5/group). (**B**) Serum levels of histone-associated DNA fragments after 1 hour of reperfusion are shown in arbitrary units (AU) of absorption in the DCD and DCD + A2 groups. (mean ± s.e.m., ** *p* < 0.01 DCD + A2 vs. DCD, Student’s t-test, *n* = 5/group).

Serum glutamate dehydrogenase (GLDH) showed similar patterns, with strongly increased serum levels on POD1 in both experimental groups and a gradual decrease throughout the observation period (Figure 4). Despite the graphical differences between the DCD and DCD + A2 groups, no significant difference was observed at any of the time-points (GLDH POD1: 574 ± 322 IU/L vs.141 ± 79 IU/L; *p =* 0.19, GLDH POD3: 375 ± 16 IU/L vs.181 ± 57 IU/L; *p =* 0.09; see Figure 3) which might be attributed to the limited sample size and relatively large variance.

Serum cAMP-dependent protein kinase activity was significantly increased following adenosine A2a receptor stimulation and 1 h of reperfusion in the samples of the DCD + A2 group compared to DCD without receptor stimulation by CGS 21680 (56% ± 3% vs. 32% ± 3%; *p* < 0.001, Figure 4).

For the detection of in situ cell apoptosis, the presence of serum low-molecular-weight histone-associated deoxyribonucleic acid (DNA)-fragments was measured 60 min after reperfusion. A significantly higher level of DNA fragmentation was observed with more than a twofold increase in the DCD group compared to the DCD + A2 animals (3.9 ± 1.0 AU vs. 1.2 ± 0.5 AU; *p* < 0.01, Figure 4).

### 2.4. Survival

**Figure 5:**
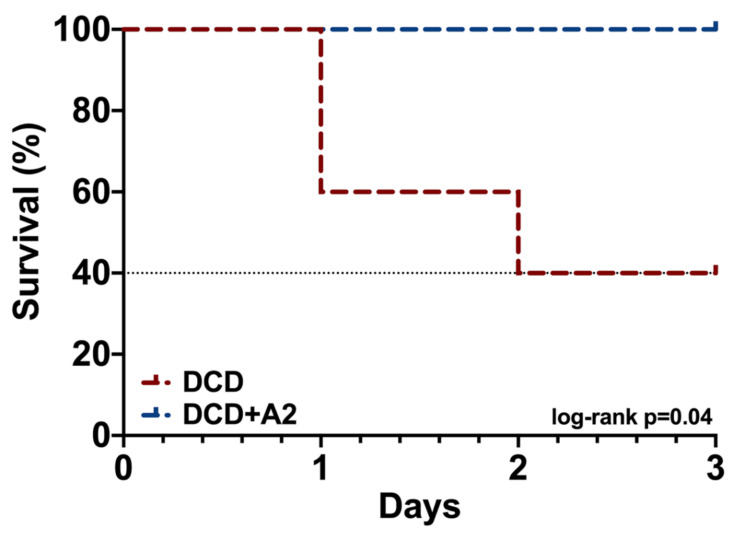
Animal survival. Kaplan–Meier analysis and log-rank test for 3-day animal survival (*p* = 0.04 log-rank test, *n* = 5/group).

Although Kaplan–Meier analysis showed a 100% (5/5) survival in the treated DCD + A2 group after three days, three animals in the DCD group were terminated due to severe DGF/PNF, resulting in a significantly reduced survival of 40% (100% vs. 40%; *p* = 0.04, see Figure 5).

## 3. Discussion

In this study, we provide in vivo evidence on the protective effects of adenosine A_2a_ receptor stimulation in a porcine model of DCD liver transplantation. Adenosine A_2a_ receptor activation by CGS 21680 led to improved functional recovery and graft microcirculation and reduced hepatocellular injury and apoptosis. Ultimately, these favorable effects were clinically manifested in better animal survival.

Although various countries (e.g., the United Kingdom, the Netherlands, Switzerland, Spain, and the United States) successfully transplant a high proportion (10–30%) of all their liver allografts following circulatory death, DCD remains a controversial issue, associated with significantly worse grafts and patient outcomes [1,28,29,30]. As the utilization of DCD and other marginal allografts is increasing continuously and is projected to increase further [31], every effort should be made to improve post-OLT outcomes using these ECD allografts [29,31]. Over the past decades, multiple strategies have been investigated along the timeline of the different steps of organ donation and transplantation, aiming to increase the ischemic tolerance of ECD allografts and improve patient outcomes [32]. This includes donor therapies, modifications of flush out and organ preservation, as well as recipient interventions [32]. Selective adenosine A_2a_ receptor stimulation has been identified as a potential cytoprotective strategy to mitigate IRI in various settings, due to its well-documented anti-inflammatory effects [22]. Recently, Lau et al. have published data on the effects of regadenoson, an adenosine A2a receptor agonist, in the setting of clinical lung transplantation [33]. In this pilot study of 14 lung transplantation recipients, the authors concluded that regadenoson can be safely infused in the setting of human lung transplantation with no dose-limiting toxicities or drug-related mortality.

Despite its positive effects, to the best of our knowledge no in vivo data is available on selective adenosine A_2a_ receptor stimulation in a large animal model of DCD liver transplantation.

During hepatic ischemia, the lack of energy substrates leads to a deterioration of active transmembrane transport mechanisms, with a consequential cell edema [34,35]. Microcirculatory failure is caused by capillary occlusion and prolonged vasoconstriction due to the diminished production of nitric oxide (NO) [1,35,36]. The reintroduction of oxygen to the liver following allograft revascularization leads to massive ROS production, which is predominantly attributed to the mitochondrial complex I [36]. Subsequently, the functional and structural damage of the mitochondria causes progressive adenosine triphosphate (ATP) depletion [34], which triggers cell death and the nuclear release of danger-associated molecular patterns (DAMPs). DAMPs in turn lead to Kupffer cell activation and release of inflammatory mediators, resulting in the migration of neutrophils into the hepatic tissue [34,37,38,39].

Because microcirculatory failure plays a key role in IRI, we monitored post-OLT liver microcirculation in our present study using laser Doppler flowmetry. Ex vivo pre-treatment on the bench with the adenosine A_2a_ agonist CGS 21680 resulted in markedly improved hepatic microcirculation following one hour of reperfusion. Hepatic microcirculation of the DCD + A2 animals was comparable with the baseline, showing significantly higher values compared to the untreated DCD group. Positive effects of adenosine A_2a_ activation on microcirculation have been demonstrated in various settings [22]. Awad et al. were able to show significantly decreased neutrophil transmigration and reduced vascular permeability in the kidney following acute IRI, which was presumably associated with the reduction of adhesion molecule expression and neutrophil adherence triggered by adenosine A_2a_ stimulation in their model [40].

In the DCD liver transplantation setting, an inevitable period of in situ warm ischemia in the donor results in a “pre-damaged” allograft with an increased susceptibility to subsequent cold preservation and reperfusion injury [41]. This cumulative damage raises the incidence of post-OLT graft dysfunction, PNF, and biliary complications [41]. Injury to the epithelial layer of the bile duct as well as of the microvascular endothelium of the peribiliary vascular plexus, along with oxidative stress, lead to a loss of the protective lining in the biliary tree, resulting in biliary complications in the long run [1,42]. Sufficient bile production is an early sign of recovery of graft function in OLT and a rapid decline in bile secretion immediately after transplantation may serve as an early marker for acute graft rejection and PNF [43]. It is also known that the higher level of hepatic ATP after an ischemic period is correlated with increased bile production [44]. In the present setting, bile production was significantly higher in the DCD + A2 group compared to the untreated control animals, suggesting an earlier functional recovery in the treated livers.

To further assess allograft function, an ICG test was performed. Indocyanine green, as a water-soluble inert compound, binds to albumin and β-lipoproteins in the blood, and is not metabolized but is almost completely excreted actively into the bile without entering the enterohepatic recirculation [45,46]. ICG-PDR is dependent on an active uptake by functional hepatocytes and its excretion from the cells; thus, it is used to assess early graft function and predicts outcomes following transplantation [47,48]. In line with the increased bile production, adenosine A_2a_ stimulation resulted in a significant improvement of PDR of the ICG dye following 1 and 3 h of reperfusion. Superior early graft function of the DCD + A2 group was confirmed further by the higher POD3 PT/Quick values compared to the untreated animals.

In the present study we aimed to assess the effects of A_2a_ receptor activation in a model of moderate to severe IRI and liver transplantation. After 30 min of circulatory death and 4.5 h of cold storage, a markedly increased hepatocellular injury was observed with a peak after 24 h of reperfusion according to the serum transaminase levels. Administration of CGS 21680 resulted in a significant reduction of tissue injury in terms of histopathological alterations, as well as serum AST and GLDH levels. In addition, the presence of apoptotic cell death was assessed by the measurement of the serum levels of histone-associated DNA fragments. Here we were able to show a more than twofold increase in DNA fragmentation in the DCD group compared to the treated DCD + A2 group. These observations support the favorable effects of adenosine A_2a_ receptor stimulation both on necrosis and apoptosis in DCD liver transplantation.

These findings are in line with previous reports in the setting of small-for-size liver transplantation and ex vivo studies on isolated livers [26,49,50]. Warm ischemia and cold preservation injury with a subsequent warm reperfusion lead to necrotic cell death associated with the lack of energy substrates and ATP depletion [49]. Nevertheless, apoptosis also plays an important role in hepatic IRI and inferior outcomes following OLT [49,51]. Tang et al. used a rat model of small-for-size OLT and demonstrated a reduction of the apoptotic index following adenosine A_2a_ receptor stimulation, which was associated with the up- and downregulation of anti- and pro-apoptotic factors, respectively [26,50]. Similar results were found by Ben-Ari et al. in an isolated rat liver model [49]. Our abovementioned observations and the findings of previous studies suggest the presence of multiple mechanisms behind the protective effects of adenosine A_2a_ receptor stimulation in IRI-induced cellular death and tissue destruction [22].

In the actual study, three out of the five non-treated animals died within the first two days following OLT due to acute graft failure, whereas all five animals survived in the DCD + A2 group. Nonetheless, all animals of the DCD group, including those which survived throughout the whole observation period, showed higher levels of hepatocellular injury and apoptosis, impaired functional graft recovery, and reduced microcirculation. Although these findings are in line with a handful of pre-clinical studies using small animal models of OLT and warm liver ischemia [20,22,27,49,50], the present study is the first to report on the in vivo effects of selective adenosine A_2a_ receptor activation on graft function and animal survival in a large animal model of DCD liver transplantation.

Although endogenous adenosine is accumulated during ischemia and is known to be an important mediator in innate protective pathways triggered by ischemic events, the effect of direct adenosine administration is limited by its short half-life and the simultaneous stimulation of all four adenosine receptor subtypes (A1, A_2a,_ A_2b_, A3) [27,52]. Despite a significant overlap in the function of the receptors, most reports suggest that cAMP- and PKA-mediated protective and anti-inflammatory and anti-apoptotic effects are predominantly associated with the activation of the A_2a_ receptors [17,22,53], which underlines the importance of using a rather selective receptor stimulation. Previous molecular biological studies confirmed that the physiological and/or artificial stimulation of the adenosine A2a receptors induces the sequential activation of adenylate cyclase and protein kinase A (PKA) [53,54]. The latter, by phosphorylating the adenosine A2a receptor, allows the G protein-mediated activation of phosphatidylinositol 3-kinase (PI3K). In turn, PI3K inhibits necrotic and apoptotic cell death via PDK-1/PKC/p38-MAPK- and PDK-1/Akt/BAD-mediated downstream pathways, respectively [53,54]. Therefore, we have measured the activity ratio of PKA in serum samples following OLT with and without adenosine A2a receptor stimulation. We demonstrated significantly increased PKA activation in the DCD + A2 group, compared to controls. This observation may be interpreted as a successful direct confirmation of the adenosine A2a receptor stimulation in our model and is in line with our descriptive findings showing less hepatocellular enzyme release, reduced necrotic and apoptotic cell death, and improved clinical outcomes.

The findings of this experimental study should be interpreted in the light of potential limiting factors. First, due to the logistical and surgical complexity of the porcine DCD OLT model, only a limited number of animals were included in our study. This may limit the translation of these findings to a larger populational level. Second, our study is partially descriptive, delivering only limited insights into the exact mechanisms by which adenosine A_2a_ receptor stimulation reduces IRI and improves outcomes in DCD OLT. In the present study, only the PKA activity ratio was measured to confirm the molecular activation of the downstream pathways potentially triggered by adenosine A2a receptor agonism. Third, due to the abovementioned logistical reasons and because of animal welfare and 3R (refining severity, as well as reducing the number of animals) considerations, the administration protocol for CGS 21680 was simply based on our previous experience and literature findings and no further experiments were performed to determine the potentially optimal dosage [17].

In conclusion, the present study delivers novel results on the potent protective effects of adenosine A_2a_ receptor activation in a clinically relevant large animal model of DCD liver transplantation. Adenosine A_2a_ receptor stimulation with CGS 21680 flush on the bench seemed to be a feasible method which was able to significantly reduce tissue injury, DNA fragmentation, and apoptosis, and improve graft microcirculation and function, in parallel with better animal survival in our setting. Based on these results, a more detailed exploration of these effects would be of interest for basic and translational research. The increasing clinical utilization of various hypothermic machine perfusion and dynamic organ preservation techniques may provide an excellent platform to facilitate the clinical translation of this and other ex vivo cytoprotective therapies [1,5,55,56,57].

## 4. Materials and Methods

### 4.1. Animals and Ethics

All experiments were performed in accordance with institutional guidelines and the German federal law regarding the protection of animals. The ethical proposal of the study was approved by the responsible Governmental Animal Care and Use Committee (Bezirksregierung Köln, Cologne, Germany, number 50.203.2-BN 45, 01.09.2006). All animals in the present study received human care according to the principles of the “Guide for the Care and Use of Laboratory Animals” (8th edition, NIH Publication, 2011, Bethesda, MD, USA) The present study has been reported according to the basic principles of the ARRIVE (Animal Research: Reporting of In Vivo Experiments) guidelines [58].

Female outbred German Landrace pigs (*n* = 10, 20–25 kg) were used as donors and recipients in an allogenic model of OLT [59]. All animals were obtained from a local disease-free breeding facility, delivered to our research facility 7 days before surgery for acclimatization and housed in a temperature- and humidity-controlled barrier environment with a 12-h light and dark cycle. The animals were fasted overnight prior to the surgical procedures. Routine laboratory testing showed normal baseline hepatic function. Orthotopic liver transplantation was conducted to a weight-matched recipient (<5 kg difference).

### 4.2. Experimental Design

**Figure 6:**
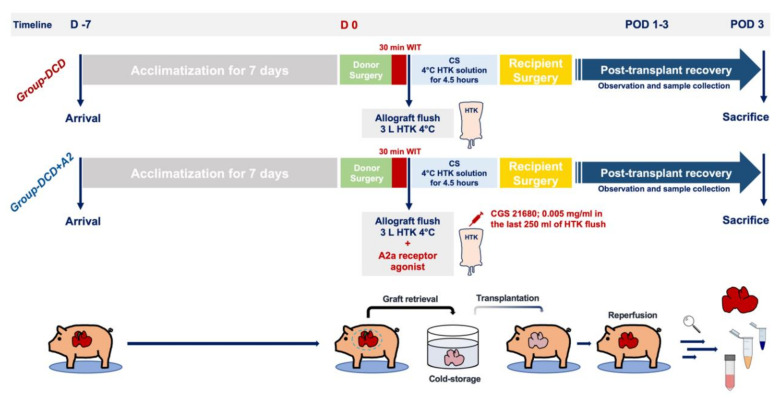
Study flowchart and surgical protocols. Animals were randomized into two experimental groups (DCD and DCD + A2). Following a 7-day acclimatization period in our housing facility, a liver graft from the donor animal was retrieved and implanted into the recipient animal following 30 min of circulatory death/in situ warm ischemia and 4.5 hours of cold preservation. Liver grafts in the DCD + A2 group were flushed with a standard dose of CGS 21680 adenosine A2a receptor agonist added to the HTK solution. Animals were monitored closely and sacrificed after 3 days of reperfusion for sample collection and further analysis (*n* = 5/group). Abbreviations used: DCD—donation after circulatory death; HTK—histidine-tryptophan-ketoglutarate solution; POD—postoperative day; CS—cold storage; WIT—warm ischemia time.

For the present study, 10 cases of whole-graft allogenic OLT procedures were performed based on an a priori sample size estimation. Animals were randomly allocated into two experimental groups (*n* = 5 cases/group) (Figure 6).

Group DCD: following electrically-induced cardiac arrest and a warm ischemia time (WIT) of 30 min in the donor, liver grafts were flushed with 3 liters of 4 °C HTK (Custodiol^®^; Dr. Franz Köhler Chemie GmbH, Bensheim, Germany) through the portal vein using a low-pressure system (<20 cm H2O).

Group DCD + A2: following electrically-induced cardiac arrest and a WIT of 30 min in the donor, liver grafts were perfused with 3 liters of 4 °C HTK, as described above. Selective A2a receptor agonist CGS 21680 (Sigma Chemicals, Saint Louis, MO, USA) was added to the last 250 mL of the HTK solution (0.005 mg/mL). The dosage for the CGS 21680 was modified for a large animal setting from previous studies by our group and by others [17,49,60].

The subsequent procedures of back table dissection, cold storage, OLT, and postoperative care were identical in both experimental groups.

### 4.3. Anesthesia, Liver Retrieval, and Adenenosin A2a Receptor Stimulation

All surgical procedures were performed under sterile conditions according to the general principles of surgical asepsis and antisepsis. Animals were premedicated with the intramuscular application of 15 mg/kg ketamine (Ratiopharm GmbH Ulm, Germany), 0.3 mg/kg xylazine (Rompun, Bayer AG, Leverkusen, Germany), and 0.1 mg/kg atropine sulphate (B. Braun Melsungen AG, Melsungen, Germany).

General anesthesia was induced by i.v. administration of 0.3 mg/kg midazolam (Ratiopharm GmbH Ulm, Germany), 0.2 mg/kg pancuronium (Ratiopharm GmbH Ulm, Germany), and 12.5 µg/kg fentanyl (Janssen-Cilag, Neuss, Germany). After endotracheal intubation, anesthesia was maintained by mechanical ventilation and isoflurane (Piramal Critical Care GmbH, Hallbergmoos, Germany) (final expiratory 1.45–2.0 Vol.%), fentanyl (3–7.5 μg/kg/h), and propofol (Fresenius Kabi GmbH, Bad Homburg, Germany) (2–4 mg/kg/h).

The right external jugular vein was cannulated using an open surgical technique and a central venous catheter (Cavafix Certo 375, B. Braun Melsungen AG, Germany) was inserted to monitor central venous pressure and for the collection of blood samples during the postoperative period. The jugular catheter was then tunneled to the back of the animal. An arterial catheter (Arterial Leadercath, Vygon, Aachen, Germany) was placed in the common carotid artery using the Seldinger technique for invasive blood pressure monitoring. All animals were monitored using a pulse-oximetry device placed on the tail and a standard electrocardiogram (ECG) (Datex-Ohmeda GmbH, Duisburg, Germany).

Following surgical disinfection, the abdomen was opened by a midline incision and the hepatic hilum with the hepatic artery, portal vein, and common bile duct were identified. Dissection began with the common hepatic artery until it was mobilized well below its bifurcation. A 12-French biliary catheter (Mac-Loc Locking Loop, Cook Medical GmbH, Baesweiler, Germany) was inserted into the common bile duct. Subsequently, the cystic duct and the common bile duct were divided. The portal vein and the infra-hepatic vena cava were dissected. The left and right triangular ligaments were divided, the organ was detached from the diaphragm, and all minor hepatic veins were ligated. Ventricular fibrillation and cardiac arrest were induced using direct electrical stimulation of the heart with a standard AAA 9 V battery, following phrenotomy, as described previously [61]. Cardiac arrest was confirmed on the ECG and followed by a 30-min “no-touch” period. During this period the ventilator was stopped, and core rectal temperature was kept at 37 °C–38 °C using a heating pad and warmed air. No heparin was used in the donor animal. Subsequently, in situ perfusion of the liver was achieved with 3 liters of 4 °C HTK solution via the portal vein at gravity (<20 cm H2O) using a portal vein cannula (Maquet Cardiopulmonary GmbH, Rastatt, Germany). In the DCD + A2 group, 0.005 mg/mL of CGS 21680 was added to the last 250 mL of the HTK-flush. The abdominal cavity was then filled with crushed ice for additional topical cooling. Subsequently, vascular structures of the liver were clamped and divided, and the allograft was carefully removed. The first 250 mL of blood, drained from the vena cava, was collected in a blood collection bag with citrate-phosphate-dextrose for on-demand transfusion in the recipient.

### 4.4. Bench Dissection and Cold Storage

Bench dissection was performed in a standard fashion [62]. The hepatic artery was flushed on the bench with 100 mL of cold HTK. The liver graft was then stored in HTK solution with a target cold ischemic time (CIT) of 4.5 h at 4 °C using an external computer-controlled cooling circuit (Haake F3, Thermo Haake GmbH, Karlsruhe, Germany).

### 4.5. Orthotopic Liver Transplantation and Postoperative Care

Anesthesia and preoperative preparation were performed as described above. Median laparotomy was performed in the same fashion, and recipient hepatectomy was completed as described [62]. Rectal temperature, ECG, arterial blood pressure, central venous pressure, and diuresis were monitored throughout the intraoperative phase. Before completing the recipient hepatectomy, an active veno-venous bypass was introduced to shunt the blood from the portal vein and the infrahepatic vena cava to the left external jugular vein using a centrifugal pump and a corresponding disposable set (Medtronic Bio-Medicus, Minneapolis, MN, USA). Anticoagulation during this period was achieved by addition of 3000 IU of heparin (Ratiopharm GmbH, Ulm, Germany) and 1 liter of 6% hydroxyethyl starch (B. Braun) to the external circuit. After the bypass was running at about 400–500 mL/min and the animal was hemodynamically stable, recipient hepatectomy was completed and the liver graft was transplanted orthotopically, as described [62]. End-to-end anastomosis of the supra- and infra-hepatic vena cava was performed with 4-0 prolene running sutures (Ethicon, Johnson and Johnson Medical GmbH, Norderstedt, Germany). The portal vein was reconstructed with a continuous anastomosis technique with 5-0 prolene sutures, followed by the hepatic artery anastomosis with 8-0 prolene interrupted sutures. Before graft reperfusion, 250 mg of prednisolon (Solu-Decortin H, Merck SeronoGmbH, Darmstadt Germany) was intravenously applied to induce immunosuppression. Before completing the portal vein anastomosis, the graft was washed with 200 mL of Ringer’s solution (B. Braun, Melsungen AG, Germany) to flush out the residual HTK solution and to remove cell detriments. In addition, the first 100 mL of blood following reperfusion was discarded before completing graft reperfusion. A portal-vein-first approach was used, and the graft was reperfused immediately after the completion of the portal anastomosis. For prophylactic stabilization of the cardiac cellular membranes, 60 mg of calcium solution in 100 mL of saline was applied i.v. immediately before portal reperfusion (CaCl 5.5%, 20 mg Ca/1 mL Baxter, Unterschleißheim, Germany). The common bile duct was externalized through the abdominal wall for later bile collection using the previously inserted biliary catheter. After checking the abdomen for bleeding, the abdomen was closed in 4 layers (peritoneum, fascia, subcutis, and skin). In the first hours following OLT, the animals were monitored closely until weaning from the respirator was completed and the animal was fully awake and was able to walk and drink spontaneously. The animals were given 1 liter of Ringer’s solution during the recovery phase over the central venous line. The animals had ad libitum access to water as soon as they could drink spontaneously. Solid food was provided from POD1. Analgesia was achieved by intramuscular tramadol (Aliud Pharma GmbH, Laichingen, Germany) injections every 6–8 h up to 72 h postoperatively. A daily dosage of 50 mg ranitidine i.v. (Ratiopharm GmbH) was also applied and prophylactic antibiotic treatment with 2 × 500 mg of ampicillin i.v., (Ratiopharm GmbH) was started prior to OLT and continued throughout the observation period. Antithrombotic prophylaxis with 500 mg of acetylsalicylic acid (Bayer Vital GmbH, Leverkusen, Germany) was given from POD1 during the whole observation period until the end of the experiment. Animals were monitored for 3 days postoperatively. All surviving animals were assessed using the human end-points score sheet by Morton and Griffiths, according to the recommendations of our team for experimental studies in the field of liver research [63,64,65,66]. Scoring and clinical evaluation of the animals were performed by an experienced veterinary technician and/or by the veterinary officer, who were not informed regarding the applied treatment or grouping to ensure a blinded assessment. Within the survival analysis, after reaching a score of ≥20 (severe strain), the animals were sacrificed and these were counted as deaths according to refinement requirements.

After POD3, animals were euthanized following the opening of the abdomen in anesthesia and sample collection. Euthanasia was performed with 10 mL T61 i.v., (200 mg Embutramid, 50 mg Mebezonium, 5 mg Tetracain; MSD Sharp and Dohme, Kenilworth, NJ, USA).

### 4.6. Enzyme Release and Prothrombine Time

Venous blood samples were collected over the jugular catheter after 1 and 3 h of reperfusion and on each morning of POD1–3. Samples were centrifuged (room temperature, 10 min, 2500 rpm) and then serum levels of AST and GLDH were measured using standard photometric procedures in an automated analyzer (Dade-Behring Dimension System, Siemens, Erlangen, Germany). PT was measured on POD3 and expressed as a percentage according to Quick’s method.

### 4.7. Bile Production and ICG-Plasma Disappearance Rate

Bile was collected during the first hour of the reperfusion period via percutaneous biliary drainage and bile production was expressed in mL/60 min.

Non-invasive pulse dye densitometry was performed using a LiMON analyzer (Pulsion Medical Systems AG, Munich Germany) and a corresponding optical finger probe. After 1 and 3 h of reperfusion, a bolus of 1 mg/kg of ICG was injected over the central line. The optical probe attached to the animal’s tail then monitored arterial ICG levels. The results reflect the amount of dye eliminated over a standard registration period of 6 min and are presented as a percentage of the initial value measured directly after injection, expressed as PDR [45].

### 4.8. Hepatic Microcirculation

Graft microcirculation was evaluated one hour after reperfusion at three standard locations on the surface of the liver using a laser Doppler monitor and corresponding surface probe (DRT4, DP1T surface probe, Moor instruments, Devon, UK). The mean value of the measurement points was used to characterize graft microcirculation. Due to the large individual variations between animals, the data were expressed as a percentage of the baseline value measured on the surface of the recipient liver before hepatectomy.

### 4.9. Serum PKA Activity Ratio

Serum PKA activity was measured in snap-frozen serum samples based on an enzyme-linked immunosorbent assay (ELISA) method that uses a synthetic PKA pseudosubstrate and a monoclonal antibody that recognizes the phosphorylated form of the peptide (Calbiochem, Bad Soden/Ts, Germany). The tests were run with and without the addition of cAMP (2.2 mmol/L) and were performed in duplicate. Thus, the activity ratio of PKA in the serum samples was evaluated by comparing the respective absorption values in the absence and/or presence of cAMP.

### 4.10. Histopathology and Apoptosis

Histological samples were harvested from identical anatomical sites of the liver before sacrifice. The excised liver was fixed in 5% neutral buffered formalin and embedded in paraffin. Slides of 10 μm in thickness were stained with hematoxylin and eosin (HE). Slides were all examined in a blinded fashion by two independent investigators, including a senior pathologist. The examining pathologists were not informed regarding the applied treatment or grouping.

Serum samples, taken 1 h after reperfusion and stored at −80 °C, were used to assess the level of low-molecular-weight (LMW) histone-associated DNA-fragments. For this, a cell death ELISA kit (Cell Death Detection ELISA, Roche Molecular Biochemicals, Frankfurt, Germany) was used. Oligonucleosome-bound DNA fragments were determined spectrophotometrically at 405 nm and presented as arbitrary units.

### 4.11. Statistics

The primary endpoint of the study was recipient survival. Among the secondary endpoints, markers of hepatocellular injury, apoptosis, graft function, and microcirculation were assessed. Results were expressed as mean ± s.e.m (standard error of the mean). Comparisons between the two experimental groups were performed using the Student’s t-test. Survival curves were generated by the Kaplan–Meier method and compared with the log-rank test. All *p*-values <0.05 were considered statistically significant. Higher levels of statistical significance (<0.01, <0.001) were used if applicable. Statistical analysis was performed using the GraphPad Prism 7 (GraphPad Software Inc., San Diego, CA, USA) software package.
